# Learning to Mitigate Epidemic Risks: A Dynamic Population Game Approach

**DOI:** 10.1007/s13235-023-00529-4

**Published:** 2023-10-21

**Authors:** Ashish R. Hota, Urmee Maitra, Ezzat Elokda, Saverio Bolognani

**Affiliations:** 1 Department of Electrical Engineering, IIT Kharagpur, Kharagpur, West Bengal 721302 India; 2 Automatic Control Laboratory, ETH Zürich, 8092 Zürich, Switzerland

**Keywords:** Dynamic population game, Epidemic mitigation, Vaccination, Testing, Perturbed best response dynamics

## Abstract

We present a dynamic population game model to capture the behavior of a large population of individuals in presence of an infectious disease or epidemic. Individuals can be in one of five possible infection states at any given time: susceptible, asymptomatic, symptomatic, recovered and unknowingly recovered, and choose whether to opt for vaccination, testing or social activity with a certain degree. We define the evolution of the proportion of agents in each epidemic state, and the notion of best response for agents that maximize long-run discounted expected reward as a function of the current state and policy. We further show the existence of a stationary Nash equilibrium and explore the transient evolution of the disease states and individual behavior under a class of evolutionary learning dynamics. Our results provide compelling insights into how individuals evaluate the trade-off among vaccination, testing and social activity under different parameter regimes, and the impact of different intervention strategies (such as restrictions on social activity) on vaccination and infection prevalence.

## Introduction

As observed during the COVID-19 pandemic, and other epidemics such as SARS-CoV-1, individuals encounter a challenging decision-making problem while attempting to protect themselves from an infectious disease. Reducing social interactions might protect them from getting infected in the short term, but comes with significant social and economic costs. While an effective vaccine may impart lasting immunity from infection, it might come at a cost and its supply may be limited as observed in developing countries such as India during COVID-19 [3, 17]. Presence of asymptomatic yet infectious agents makes the problem even more challenging as an individual is not aware of its true infection state and it may be necessary to undergo testing to detect whether one is indeed infected [2]. While it is socially desirable for individuals to undergo testing and isolate themselves if found to be infected to prevent further infections, an individual who is not at a risk of developing severe symptoms may avoid testing since isolation may cause significant mental and economic stress. Finally, although human decision-making is strategic and forward-looking, it may still suffer from a certain degree of bounded rationality, and needs to rely on a learning process while exploring the trade-off among above actions (level of social interaction, vaccination or testing). In this work, we propose a dynamic population game model to capture, in a rigorous and principled manner, how a large population of self-interested individuals take decisions regarding social activity level, vaccination and testing to protect themselves from an infectious disease.

Game theory presents a natural framework to examine decision-making by a large number of strategic decision-makers. Past work has indeed examined game-theoretic decision-making in the context of epidemics; see [9, 29] for recent reviews. For the class of susceptible-infected-susceptible (SIS) epidemic model, formulations based on single-shot or static games [26, 49], repeated games [16, 27, 48] and dynamic games [28] have been examined. Recent papers that appeared after the onset of the COVID-19 pandemic have largely focused on the susceptible-infected-recovered (SIR) epidemic model and its variants and have analyzed individual decisions to adopt social distancing measures or vaccination, albeit separately [4, 5, 27, 34]. Most of the above settings consider single-shot decision-making by the agents. For instance, [5, 26, 49] assume that vaccination decisions are made before the outbreak with infection risk given by the likelihood of becoming infected in the steady-state of the epidemic dynamics. However, as observed during COVID-19, vaccines are not necessarily available at the onset of a new epidemic, and individuals decide whether to vaccinate or not concurrent to the outbreak which makes the problem challenging and interesting to investigate. In addition, most of the above works do not consider forward-looking agents, i.e., agents do not incorporate the impact of their decisions on future state and payoffs.

Another stream of research have explored the notion of mean-field games in the context of epidemics [44], particularly for social distancing [10, 43], vaccination [15, 45] and Stackelberg game settings [7, 31]. In addition, authors in [42] examined the impact of asymptomatic infections in a partially observed mean-field game framework. However, to the best of our knowledge, past work has not explored the case where agents can choose among vaccination, social distancing and testing, each having a different impact on the state transition of the agent in an epidemic model with asymptomatic infections. In addition, computing equilibrium strategies is often challenging in this class of games. While the problem of learning in mean-field games has been studied in the past [35], this aspect has not been explored in the context of epidemics. While some works have studied evolutionary learning in the context of epidemic games in recent past [33, 36, 37, 47], these settings consider agents that are myopic rather than forward-looking.

In this work, we aim to address the above research gap and examine the behavior of a large population of far-sighted agents as they strategically choose among vaccination, testing and social activity level, and adapt their strategies following an evolutionary learning process. We build upon the recent preliminary work [18] (by a subset of the authors of this paper) and consider the susceptible-asymptomatic-infected-recovered-unknowingly recovered (SAIRU) epidemic model. This model has also been examined in the context of state estimation and prediction of epidemics in the recent past [2, 41].

**Fig. 1:**
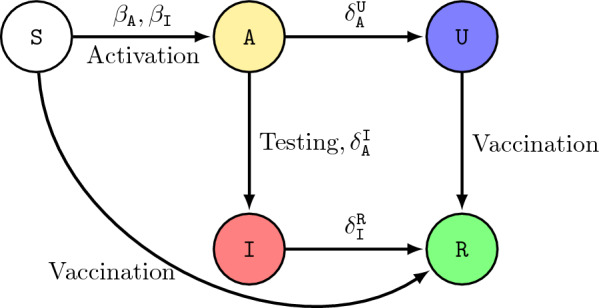
Evolution of states in the SAIRU epidemic model under activation, testing and vaccination. Self loops are omitted for better readability

In our model, at each time instant, each agent chooses whether to activate and if so how many other agents it wishes to interact with, or whether to vaccinate itself, or whether to undergo testing (see Sect. 2.2 for a formal definition). Activation comes with a reward that increases with activation degree, and a cost that captures social restrictions imposed by authorities. Similarly vaccination, testing and being symptomatically infected comes with a certain cost. Decision to vaccinate or undergo testing does not guarantee that the agent is able to obtain the vaccine or testing kit due to limited supply. Successful vaccination results in susceptible and unknowingly recovered agents developing immunity or becoming aware of their immunity and moving to the recovered state, while an asymptomatic agent undergoing testing moves to the symptomatic compartment. If an agent opts for testing in a state other than asymptomatic, its state does not change (see Fig. 1 for possible state transitions under different actions). Agents maximize a discounted infinite horizon expected reward which is a function of the infection state distribution and the policy followed by the population.

Similar to [18], we leverage the framework of *dynamic population games* proposed in [19] which is a generalization of the classical population game setting [46] to capture dynamically changing population distribution and non-myopic decision-making. Specifically, the authors in [19] show that this class of games can be reduced to a static population game setting which enables a plethora of evolutionary learning models to be applied to study evolution of user behavior in the same time-scale as population evolution. This is in contrast with mean-field games [23, 30, 39] and other models of large population stochastic games [1, 32] where it is often challenging to apply evolutionary learning strategies.

This manuscript differs from the preliminary work [18] as follows. First, in the prior work [18], agents could only choose their activation degree; vaccination and testing were not considered at all. Second, agents in asymptomatic and unknowingly recovered states were constrained to behave as if they were susceptible irrespective of the relative proportions of agents in these states. We explicitly take into account the proportion of agents in each of the above three states while defining the expected reward and best response in this work.

The contributions and structure of this paper is described below. The dynamic model of the infectious disease is presented in Sect. 2 which describes the probability of state transition for different choice of actions by the agents. The strategic decision-making process of the agents is described in Sect. 3. Section 4 defines the notion of best response and stationary Nash equilibrium for our setting followed by showing its existence. The perturbed best response dynamics to update the policy of the agents in a comparable time-scale as the state distribution update is presented in Sect. 5. Section 6 presents detailed numerical results on the evolution of the epidemic as well as the policies of the agents under the perturbed best response dynamics. We thoroughly examine the impacts of (i) vaccination cost and availability limits, (ii) myopic vs. far-sighted decision-making, and (iii) response of the population under different lockdown strategies, and observe several counterintuitive phenomena that provide critical insights for policymakers. For instance, we show that reducing cost of vaccination without increasing supply may lead to a higher peak infection level as individuals would opt for vaccination (instead of testing resulting in inadequate isolation of asymptomatic individuals).

## Epidemic Model

We consider a homogeneous population of (non-atomic) agents or individuals. The state and the dynamics of this population are described below.

### States

We consider the SAIRU epidemic model where each agent is in one of the following infection states: Susceptible (${\texttt{S}}$), Asymptomatically infected (${\texttt{A}}$), Infected with awareness (${\texttt{I}}$), Recovered (${\texttt{R}}$), and Unknowingly recovered (${\texttt{U}}$). State ${\texttt{U}}$ corresponds to agents that have recovered without ever showing symptoms. Specifically, agents in state ${\texttt{I}}$ move to state ${\texttt{R}}$ after recovery while agents in state ${\texttt{A}}$ move to state ${\texttt{U}}$ after recovery. In this work, we assume that agents in states ${\texttt{R}}$ and ${\texttt{U}}$ are immune from further infection. Formally, we define the state of an agent as $s \in {\mathcal {S}}$, where . The state distribution is $d \in {\mathcal {D}}:= \Delta ({\mathcal {S}})$, where $\Delta (X)$ is the space of probability distributions supported on *X*. We write *d*[*s*] to denote the proportion of agents with infection state *s*. Consequently, for every $s \in {\mathcal {S}}$, $d[s] \in [0,1]$ and $\sum _{s \in {\mathcal {S}}} d[s] = 1$. We now describe the actions available to the agents, and the impact of their chosen action on state transitions.

### Actions and Policies

At each time step (which could potentially represent one day), each agent strategically chooses its action  where

- $a = a_v$ denotes that the agent has decided to vaccinate itself,
- $a = a_t$ denotes that the agent has decided to get tested to determine its infection status, and
denotes the number of other agents it chooses to interact with.

The action $a=0$ signifies that the agent chooses not to interact with any other agent, i.e., it completely isolates itself during that time interval. An individual in states ${\texttt{S}}$ and ${\texttt{U}}$ upon successful vaccination moves to state ${\texttt{R}}$, i.e., it acquires immunity from future infection and is aware of its immunity status. If an agent in any other state chooses vaccination, it remains in its current state. Similarly, an individual in state ${\texttt{A}}$ moves to state ${\texttt{I}}$ upon successful testing. For all other infection states, testing does not lead to any state transition. In particular, since agents in states ${\texttt{S}}$, ${\texttt{U}}$ or ${\texttt{R}}$ are not infected and otherwise healthy, their test result would be negative.

A (Markovian) policy is denoted by $\pi : {\mathcal {S}}\rightarrow \Delta ({\mathcal {A}})$, and it maps an agent’s state $s \in {\mathcal {S}}$ to a randomization over the actions $a \in {\mathcal {A}}$. The set of all possible policies is denoted by $\Pi \subseteq \Delta ({\mathcal {A}})^{|{\mathcal {S}}|}$. In particular, $\pi [a \mid s]$ is the probability that an agent chooses action *a* when in infection state *s*. All agents are homogeneous and follow the same policy $\pi$. Policies need to be consistent with the information structure of the problem. Thus, we assume that agents that have never shown symptoms nor vaccinated successfully, and hence unaware of whether they are susceptible, asymptotically infected or unknowingly recovered, act in the same way, i.e., $\pi [\cdot \mid {\texttt{S}}] = \pi [\cdot \mid {\texttt{A}}] = \pi [\cdot \mid {\texttt{U}}]$.

The concatenation of the policy and state distribution is the *social state* $(\pi ,d) \in \Pi \times {\mathcal {D}}$. This gives a complete macroscopic description of the distribution of the agents’ states, as well as how they behave. The social state $(\pi ,d)$ is a time-varying quantity. In the following subsection, we describe how the state distribution evolves as a function of the current state and policy. In Sect. 5, we discuss how the policy evolves in time (denoting the learning process of the agents).

#### Remark 1

We clarify that at a given time step, an agent can either choose its activation degree or vaccination or testing. If we allow for multiple actions to be chosen at the same time, it would make the model unnecessarily complex and the dimension of the set of feasible actions will increase in a combinatorial manner. Since testing and vaccination are conducted in a controlled environment such as a hospital, we assume that appropriate social distancing behavior is followed and the likelihood of new infection is small when those actions are chosen. We emphasize that the actions are not binding beyond one time step and an agent is always free to choose a different action in the next time step if it is optimal to do so.

### State Transitions

We now derive a dynamic model of the evolution of state distribution *d* when the agents adopt a policy $\pi$. The state of each agent changes at every time step according to transition probabilities encoded by the *stochastic matrix*

1 $$\begin{aligned} P[s^+ \mid s](\pi ,d) = \sum _{a \in {\mathcal {A}}} \pi [a \mid s] \, {\mathbb {P}}[s^+ \mid s,a](\pi ,d), \end{aligned}$$

where ${\mathbb {P}}[s^+ \mid s,a](\pi ,d)$ denotes the probability distribution over the next state when an agent in infection state *s* chooses action *a* in social state $(\pi ,d)$. Note that the Markov chain $P[s^+ \mid s](\pi ,d)$ is not time-homogeneous as the social state $(\pi ,d)$ is time-varying. However, it becomes time-homogeneous if $(\pi ,d)$ is stationary; we explore this stationary regime in Sect. 4.

In order to define the state transition probabilities ${\mathbb {P}}[s^+ \mid s,a](\pi ,d)$ for different state-action pairs, we combine the transition rules of the epidemic model with the specific actions as described next. A schematic of the state transitions under different actions is given in Fig. 1.

#### State Transitions for Susceptible Agents

We first consider state transitions due to social interactions. At a given time, an agent in state *s* chooses its activation degree  according to policy $\pi$. Then, it is paired randomly with up to *a* other individuals with the probability of being connected with another agent being proportional to the activation degree of the target agent (analogous to the configuration model [40]). The agent could also fail to pair with one or more of the *a* other individuals. This occurs with increasing probability as the *total amount of activity* is low. This represents, for example, when the public space (streets, buildings) are largely empty because most agents are staying at home.

Once the network is formed, a susceptible agent becomes asymptomatically infected with probability $\beta _{\texttt{A}}\in (0,1)$ for each asymptomatic neighbor and with probability $\beta _{\texttt{I}}\in (0,1)$ for each infected neighbor. Following [6, 21], we assume that the new infection always starts in the asymptomatic state. We now formally define the transition probabilities starting from susceptible state. We first define the *total amount or mass of activity* at social state $(\pi ,d)$ as

$$\begin{aligned} e(\pi ,d) = \sum _{s \in {\mathcal {S}}} d[s] \, \sum _{a \in \{0,1,\ldots ,{a_{\text {max}}}\}} a \, \pi [a \mid s], \end{aligned}$$

which is determined by the mass of active agents and their chosen degrees of activation under policy $\pi$. Similarly, the mass of activity by *asymptomatic* and *symptomatic* agents are

$$\begin{aligned} e^{\texttt{A}}(\pi ,d)&= d[{\texttt{A}}] \, \sum _{a \in \{0,1,\ldots ,{a_{\text {max}}}\}} a \, \pi [a \mid {\texttt{A}}], \quad e^{\texttt{I}}(\pi ,d) = d[{\texttt{I}}] \, \sum _{a \in \{0,1,\ldots ,{a_{\text {max}}}\}} a \, \pi [a \mid {\texttt{I}}]. \end{aligned}$$

In order to consider the event of failing to pair with an agent when the amount of activity $e(\pi ,d)$ is low, we introduce a small constant amount $\epsilon > 0$ of fictitious activation that does not belong to any of the agents. Consequently, the probability of not interacting with any agent, the probability of a randomly chosen agent being asymptomatic and the probability of a randomly chosen agent being symptomatic are, respectively,

2 $$\begin{aligned} \gamma ^\emptyset (\pi ,d)&= \frac{\epsilon }{e(\pi ,d) + \epsilon }, \quad \gamma ^{\texttt{A}}(\pi ,d) = \frac{e^{\texttt{A}}(\pi ,d)}{e(\pi ,d) + \epsilon }, \quad \gamma ^{\texttt{I}}(\pi ,d) = \frac{e^{\texttt{I}}(\pi ,d)}{e(\pi ,d) + \epsilon }. \end{aligned}$$

Note that for a given $\epsilon$, the probability of encountering an infected agent (symptomatically or not) goes to zero as the amount of infections goes to zero, as desired. As a result, the probability of a susceptible agent to *not* get infected upon activation with degree *a* is

$$\begin{aligned} {\mathbb {P}}[s^+= {\texttt{S}}\mid s = {\texttt{S}},a](\pi ,d) =\left( 1 - \beta _{\texttt{A}}\, \gamma ^{\texttt{A}}(\pi ,d) - \beta _{\texttt{I}}\, \gamma ^{\texttt{I}}(\pi ,d)\right) ^a. \end{aligned}$$

It is easy to see that when a susceptible agent does not interact with any other agent (i.e., $a=0$), it remains susceptible. We define $0^0=1$ for the special case $1 - \beta _{\texttt{A}}\, \gamma ^{\texttt{A}}(\pi ,d) - \beta _{\texttt{I}}\, \gamma ^{\texttt{I}}(\pi ,d) = 0$. When this agent participates in exactly one interaction ($a=1$), the probability that its neighbor is asymptomatic (respectively, symptomatically infected) is $\gamma ^{\texttt{A}}(\pi ,d)$ (respectively, $\gamma ^{\texttt{I}}(\pi ,d)$). When it draws $a>0$ independent agents to interact with, it must not get infected in any of the interactions to remain susceptible, and this occurs with the probability specified above. As a consequence, we have

$$\begin{aligned}&{\mathbb {P}}[s^+= {\texttt{A}}\mid s = {\texttt{S}},a](\pi ,d) = 1 - {\mathbb {P}}[s^+= {\texttt{S}}\mid s = {\texttt{S}},a](\pi ,d). \end{aligned}$$

If a susceptible agent decides to undergo testing, it remains in susceptible state. If it chooses to vaccinate itself, it transitions to knowingly recovered state (${\texttt{R}}$) as it becomes immune to future infection. However, as observed in several developing countries during COVID-19, the number of vaccines that are available on a given day is finite and potentially much smaller compared to the total population [3, 17]. Let the proportion of individuals that can be vaccinated on a given time period be $v_{\max }$, i.e., $v_{\max }$ is the vaccine availability limit. Let $v(\pi ,d) = \sum _{s\in {\mathcal {S}}} d[s]\pi [a_v\mid s]$ denote the total mass of agents who opt for vaccination at social state $(\pi ,d)$. The transition probability from susceptible to recovered can now be defined as

3 $$\begin{aligned} {\mathbb {P}}[s^+= {\texttt{R}}\mid s = {\texttt{S}},a_v](\pi ,d) = {\left\{ \begin{array}{ll} 1, \qquad &  \text {if} \quad v(\pi ,d) \le v_{\max }, \\ \frac{v_{\max }}{v(\pi ,d)}, \qquad &  \text {otherwise}. \end{array}\right. } \end{aligned}$$

Settings with sufficient supply of vaccines can be easily modeled by setting $v_{\max }=1$. The probabilities of remaining susceptible upon choosing testing and vaccination are

$$\begin{aligned}&{\mathbb {P}}[s^+= {\texttt{S}}\mid s = {\texttt{S}},a_t] = 1, \\  &{\mathbb {P}}[s^+= {\texttt{S}}\mid s = {\texttt{S}},a_v] = 1 - {\mathbb {P}}[s^+= {\texttt{R}}\mid s = {\texttt{S}},a_v], \end{aligned}$$

where $(\pi ,d)$ is omitted for brevity of notation. While the number of available tests is also limited, it does not affect the transition of susceptible individuals who remain susceptible irrespective of whether they were able to get tested or not.

#### State Transitions for Asymptomatic Agents

If an asymptomatic agent chooses not to undergo testing, it transitions to state ${\texttt{I}}$ with probability $\delta _{\texttt{A}}^{\texttt{I}}\in (0,1)$ which represents aggravation of the illness leading to the agent developing symptoms. An asymptomatic agent may also recover without being aware of ever being infected (i.e., transitions from state ${\texttt{A}}$ to state ${\texttt{U}}$) with probability $\delta _{\texttt{A}}^{\texttt{U}}\in (0,1)$. If the agent fails to recover or develop symptoms or does not undergo testing, then it remains in asymptomatic state. Thus for $a \ne a_t$, we define the following state transition probabilities:

$$\begin{aligned}&{\mathbb {P}}[s^+ = {\texttt{U}}\mid s = {\texttt{A}}, a] = \delta _{\texttt{A}}^{\texttt{U}}, \\  &{\mathbb {P}}[s^+ = {\texttt{I}}\mid s = {\texttt{A}}, a] = (1-\delta _{\texttt{A}}^{\texttt{U}})\delta _{\texttt{A}}^{\texttt{I}}, \\  &{\mathbb {P}}[s^+ = {\texttt{A}}\mid s = {\texttt{A}}, a] = (1-\delta _{\texttt{A}}^{\texttt{U}})(1 - \delta _{\texttt{A}}^{\texttt{I}}). \end{aligned}$$

If an agent chooses to undergo testing (i.e., chooses $a=a_t$), then it becomes aware of its infection status and transitions to state ${\texttt{I}}$ depending on total availability of testing kits $t_{\max }$, and the mass of individuals that opt to get tested $t(\pi ,d) = \sum _{s\in {\mathcal {S}}} d[s]\pi [a_t\mid s]$ at social state $(\pi ,d)$. It remains in the asymptomatic state if it neither recovers, develops symptoms on its own or fails to get tested. Therefore, we have

$$\begin{aligned}&{\mathbb {P}}[s^+ = {\texttt{U}}\mid s = {\texttt{A}}, a_t] = \delta _{\texttt{A}}^{\texttt{U}}, \\  &{\mathbb {P}}[s^+= {\texttt{I}}\mid s = {\texttt{A}},a_t] = 1 - {\mathbb {P}}[s^+ = {\texttt{U}}\mid s = {\texttt{A}}, a_t] - {\mathbb {P}}[s^+= {\texttt{A}}\mid s = {\texttt{A}},a_t], \end{aligned}$$

where

4 $$\begin{aligned} {\mathbb {P}}[s^+= {\texttt{A}}\mid s = {\texttt{A}},a_t] = {\left\{ \begin{array}{ll} (1-\delta _{\texttt{A}}^{\texttt{U}})(1-\delta _{\texttt{A}}^{\texttt{I}})\left( 1 - \frac{t_{\max }}{t(\pi ,d)}\right) , \qquad &  \text {if} \quad t(\pi ,d) \ge t_{\max }, \\ 0 \quad &  \text {otherwise}. \end{array}\right. } \end{aligned}$$

In particular, when $t(\pi ,d) \le t_{\max }$, any agent who opts for testing is successful in obtaining one, and if it happens to be asymptomatic, ceases to remain in that state. It either recovers with probability $\delta _{\texttt{A}}^{\texttt{U}}$ or becomes symptomatic with probability $1-\delta _{\texttt{A}}^{\texttt{U}}$. The dependence of the above transition probabilities on $(\pi ,d)$ is omitted for brevity of notation.

#### State Transitions for Infected Agents

An infected agent in state ${\texttt{I}}$ recovers and moves to state ${\texttt{R}}$ with probability $\delta _{\texttt{I}}^{\texttt{R}}\in (0,1)$ irrespective of its action. Otherwise, it remains infected. Formally, for any $a \in {\mathcal {A}}$,

$$\begin{aligned} {\mathbb {P}}[s^+ = {\texttt{R}}\mid s = {\texttt{I}}, a] = \delta _{\texttt{I}}^{\texttt{R}}, \quad {\mathbb {P}}[s^+ = {\texttt{I}}\mid s = {\texttt{I}}, a] = 1-\delta _{\texttt{I}}^{\texttt{R}}. \end{aligned}$$

#### State Transitions for Recovered Agents

Similarly, an agent in state ${\texttt{R}}$ does not get infected again and remains in this state irrespective of control action. Consequently, for every $a \in {\mathcal {A}}$, we have

$$\begin{aligned} {\mathbb {P}}[s^+ = {\texttt{R}}\mid s = {\texttt{R}}, a] = 1. \end{aligned}$$

#### State Transitions for Unknowingly Recovered Agents

An agent in state ${\texttt{U}}$ does not get infected again. However, it is not aware of its immunity status unless it vaccinates. We therefore assume that upon successful vaccination it becomes aware of its immunity and moves to state ${\texttt{R}}$, otherwise it remains in state ${\texttt{U}}$. Consequently, we have

5 $$\begin{aligned} {\mathbb {P}}[s^+= {\texttt{R}}\mid s = {\texttt{U}},a_v](\pi ,d) = {\left\{ \begin{array}{ll} 1, \qquad &  \text {if} \quad v(\pi ,d) \le v_{\max } \\ \frac{v_{\max }}{v(\pi ,d)}, \qquad &  \text {otherwise}, \end{array}\right. } \end{aligned}$$

and

$$\begin{aligned}&{\mathbb {P}}[s^+ = {\texttt{U}}\mid s = {\texttt{U}}, a] = 1 \quad \text {if} \quad a \ne a_v, \quad \text {and} \\  &{\mathbb {P}}[s^+ = {\texttt{U}}\mid s = {\texttt{U}}, a_v](\pi ,d) = 1 - {\mathbb {P}}[s^+ = {\texttt{R}}\mid s = {\texttt{U}}, a_v](\pi ,d). \end{aligned}$$

Once the state transitions take place, the network gets discarded at the next time step and the process repeats. Note that we do not consider possibility of reinfection, i.e., loss of immunity after recovery or vaccination, in this work. There are indeed several infectious diseases, such as SARS-CoV-1, where recovery from the disease imparts immunity from further infection for a long period of time [51], and even COVID-19 imparts immunity for a few months after vaccination or infection. We discuss the technical challenges associated with including loss of immunity in our model in Sect. 7 and motivate this as a promising direction for further research. We now describe the decision-making process of the agents in the following section.

## Strategic Decision-Making by Agents

In this work, we consider agents who aim to maximize long-run discounted rewards by learning suitable policies. We first define the immediate or stage reward obtained by an agent for different disease states and chosen actions. We then define the value function for an agent in a manner that is consistent with the information structure defined earlier, and define the notion of best response and stationary Nash equilibrium.

### Rewards

Each agent derives an immediate reward as a function of its state and action composed of a reward ${r_{\text {act}}}[s,a]$ for its activation decision, a reward ${r_{\text {vax}}}[a]$ for its vaccination decision, a reward for the decision to undergo testing ${r_{\text {test}}}[a]$, and a reward ${r_{\text {dis}}}[s]$ for how its health is affected by the disease. Formally,

6 $$\begin{aligned} r[s,a]:= {r_{\text {act}}}[s,a] + {r_{\text {vax}}}[a] + {r_{\text {test}}}[a] + {r_{\text {dis}}}[s]. \end{aligned}$$

We now specify each component of the reward function. The activation reward is defined as

$$\begin{aligned} {r_{\text {act}}}[s,a]:= o[a] - c[s, a], \end{aligned}$$

where $o[a] \in \mathbb {R}_{+}$ denotes the social benefit of interacting with *a* other agents and is assumed to be non-decreasing in *a* for  with $o[0] = o[a_v] = o[a_t] = 0$. The cost $c[s,a] \in \mathbb {R}_{+}$ denotes the cost imposed by the authorities to discourage social interaction and is assumed to be non-decreasing in *a* with

$$\begin{aligned} \!\!\!c[{\texttt{I}},a] \ge c[{\texttt{S}},a] = c[{\texttt{A}},a] = c[{\texttt{U}},a] \ge c[{\texttt{R}},a] \end{aligned}$$

element-wise. The above assumption is quite versatile and allows us to model a variety of (infection state-dependent) interventions such as (a) imposing more stringent restrictions on symptomatically infected individuals, (b) exempting agents in state ${\texttt{R}}$ from social distancing restrictions (e.g., by setting $c[{\texttt{R}},a]=0$), and (c) discouraging large gatherings by setting $c[{\texttt{S}},a]$ to a very high value for *a* larger than a permissible limit. We examine the impacts of such choice of cost parameters in our numerical results in Sect. 6.

An agent who decides to vaccinate incurs a cost ${c_{\text {vax}}}$, and accordingly, we define

$$\begin{aligned} {r_{\text {vax}}}[a]:= {\left\{ \begin{array}{ll} 0 &  \text {if } a \ne a_v, \\ -{c_{\text {vax}}}&  \text {if } a = a_v. \end{array}\right. } \end{aligned}$$

The cost of vaccination could potentially reflect the hesitation among some individuals to opt for a newly developed vaccine for fear of adverse reaction, being influenced by conspiracy theories, being skeptical about the effectiveness of the vaccine or the time and efforts required in obtaining a vaccine (as observed in some developing countries during COVID-19 [3, 14]).

Similarly, an agent who decides to undergo testing incurs a cost ${c_{\text {test}}}$ leading to

$$\begin{aligned} {r_{\text {test}}}[a]:= {\left\{ \begin{array}{ll} 0 &  \text {if } a \ne a_t, \\ -{c_{\text {test}}}&  \text {if } a = a_t. \end{array}\right. } \end{aligned}$$

The final term in (6) encodes the cost of being ill:

$$\begin{aligned} {r_{\text {dis}}}[s]:= {\left\{ \begin{array}{ll} - {c_{\text {dis}}}&  \text {if }s = {\texttt{I}}, \\ 0 &  \text {otherwise}. \end{array}\right. } \end{aligned}$$

The cost ${c_{\text {dis}}}$ encodes both the cost of being severely ill and possibly also the cost of being isolated/quarantined for the duration of the disease. All three parameters ${c_{\text {vax}}}, {c_{\text {test}}}, {c_{\text {dis}}}$ are assumed to be positive constants.

It follows from the above discussion that for any specific action $a \in {\mathcal {A}}$, the stage reward for agents in susceptible, asymptomatic and unknowingly recovered states coincide, i.e., $r[{\texttt{S}},a] = r[{\texttt{A}},a] = r[{\texttt{U}},a]$.

### Effective Transition Probabilities

In order to analyze the discounted infinite-horizon expected reward, we borrow terminologies from the Markov decision process (MDP) and dynamic programming literature. We first define the expected reward, value function and single-stage deviation value (*Q*) function under a given social state $(\pi ,d)$. Subsequently, we discuss the evolution of this social state under evolutionary learning algorithms.

Note that an agent in states  is unaware of its exact infection state. From the discussion in the previous subsection, we also note that the stage reward for such an agent is identical regardless of its exact infection state. However, the long-run expected reward depends on the probability of transition from this set to other known infection states, namely ${\texttt{I}}$ and ${\texttt{R}}$. Unfortunately, these transitions depend on the exact infection state; in particular, only asymptomatic agents transit to ${\texttt{I}}$ state (upon testing or developing symptoms) and only non-infected agents transit to ${\texttt{R}}$ state upon vaccination.

In order to tackle this challenge, we assume that each agent is aware of the proportion of agents in states , and assumes that it belongs to each of the above three states with a probability that coincides with the proportion of agents in that state. While it could be challenging to accurately estimate the proportion of agents in different infection states, we believe that the agents likely have a reasonable idea regarding these proportions due to availability of testing data and outcome of serology tests. In addition, these proportions may be estimated as discussed below.

- At the onset of an epidemic, only a tiny fraction of the population is infected and almost none of the population has lasting immunity (as was the case with COVID-19). Therefore, it is reasonable to assume that agents are aware of the initial proportion of agents in states  up to a close approximation. If the initial state distribution is approximately known, the subsequent state distribution can be obtained relatively accurately by updating the state distribution under the transition law stated in equation (1) at the currently adopted policy at every instant.
- Agents may maintain a belief about their current infection state (which could correspond to a *Hidden Markov Model* (HMM) estimate) from observed states ${\texttt{I}}$ and ${\texttt{R}}$; in particular by repeatedly updating a *belief state* vector (which consists of the probability of the agent being in states ${\texttt{S}}, {\texttt{A}}$ and ${\texttt{U}}$, respectively) using Bayes’ theorem. Since we consider a large population regime, the belief state vector can then be interpreted as the proportion of agents in states ${\texttt{S}}, {\texttt{A}}$ and ${\texttt{U}}$.

Let $\widehat{d}$ denote the belief of the agents about the state distribution at a given time instant. While we assume $\widehat{d} = d$ for some of our results, we use a distinct notation here to make the distinction precise and avoid confusion. We define a super state  and define the state transition probability matrix, denoted $\widehat{{\mathbb {P}}}[s^+|s,a](\pi ,\widehat{d})$ among states ${\texttt{T}}, {\texttt{I}}$ and ${\texttt{R}}$ at social state $(\pi ,\widehat{d})$. These transition probabilities capture the belief formed by an agent regarding its next state as a function of its action, current known infection state and its belief $\widehat{d}$.

Since agents in states ${\texttt{I}}$ and ${\texttt{R}}$ are aware of their exact infection state, state transitions among them coincide with the definitions given earlier, i.e., for every $a \in {\mathcal {A}}$,

$$\begin{aligned} \widehat{{\mathbb {P}}}[s^+ = {\texttt{R}}\mid s = {\texttt{I}}, a]&= {\mathbb {P}}[s^+ = {\texttt{R}}\mid s = {\texttt{I}}, a] = \delta _{\texttt{I}}^{\texttt{R}}, \\ \widehat{{\mathbb {P}}}[s^+ = {\texttt{I}}\mid s = {\texttt{I}}, a]&= {\mathbb {P}}[s^+ = {\texttt{I}}\mid s = {\texttt{I}}, a] = 1-\delta _{\texttt{I}}^{\texttt{R}}, \\ \widehat{{\mathbb {P}}}[s^+ = {\texttt{R}}\mid s = {\texttt{R}}, a]&= {\mathbb {P}}[s^+ = {\texttt{R}}\mid s = {\texttt{R}}, a] = 1. \end{aligned}$$

The above transition probabilities are independent of $(\pi ,\widehat{d})$.

An agent in state ${\texttt{T}}$ moves to state ${\texttt{R}}$ if it is either susceptible or unknowingly recovered and chooses to vaccinate itself. Given the current belief $\widehat{d}$, this transition probability is given by

$$\begin{aligned} \widehat{{\mathbb {P}}}[s^+ = {\texttt{R}}\mid s = {\texttt{T}}, a](\pi ,\widehat{d}) = {\left\{ \begin{array}{ll} \frac{\max (\widehat{d}[{\texttt{S}}],\epsilon ) + \widehat{d}[{\texttt{U}}] }{\max (\widehat{d}[{\texttt{S}}], \epsilon ) + \widehat{d}[{\texttt{A}}] + \widehat{d}[{\texttt{U}}] } \frac{\min (v(\pi ,\widehat{d}),v_{\max }) }{v(\pi ,\widehat{d}) }, \quad &  \text {if} \quad a = a_v, \\ 0, \quad &  \text {otherwise}, \end{array}\right. } \end{aligned}$$

where $v_{\max }$ captures vaccine availability and $v(\pi ,\widehat{d})$ denotes the total mass of individuals who opt for vaccination as defined earlier. In particular, the second term denotes the probability of being successful in getting vaccinated and the first term denotes the probability of recovery upon successful vaccination. The parameter $\epsilon$ prevents discontinuity at the corner case where $\widehat{d}[{\texttt{T}}] = 0$. Essentially, we assume that the belief on the proportion of susceptible individuals is non-zero and at least a small constant $\epsilon$.

Similarly, an agent in state ${\texttt{T}}$ moves to state ${\texttt{I}}$ only when it is asymptomatic and either undergoes testing or develops symptoms on its own. This transition probability is given by

$$\begin{aligned} \widehat{{\mathbb {P}}}[s^+ = {\texttt{I}}\mid s = {\texttt{T}}, a](\pi ,\widehat{d}) = \frac{\widehat{d}[{\texttt{A}}]}{\max (\widehat{d}[{\texttt{S}}], \epsilon ) + \widehat{d}[{\texttt{A}}] + \widehat{d}[{\texttt{U}}] } {\mathbb {P}}[s^+ = {\texttt{I}}\mid s = {\texttt{A}}, a](\pi ,\widehat{d}), \end{aligned}$$

where the first term denotes the belief of the concerned agent that it is asymptomatic and the second term is the probability of an asymptomatic agent becoming infected at social state $(\pi ,\widehat{d})$ defined earlier. Finally, we also have

$$\begin{aligned} \widehat{{\mathbb {P}}}[s^+ = {\texttt{T}}\mid s = {\texttt{T}}, a] = 1 - \widehat{{\mathbb {P}}}[s^+ = {\texttt{I}}\mid s = {\texttt{T}}, a] - \widehat{{\mathbb {P}}}[s^+ = {\texttt{R}}\mid s = {\texttt{T}}, a]. \end{aligned}$$

### Expected Discounted Reward

We now define the long-run discounted expected reward for each epidemic state. First, observe that the immediate expected reward of an agent in state  when it follows policy $\pi$ is

$$\begin{aligned} R[s](\pi ) = \sum _{a \in {\mathcal {A}}} \pi [a \mid s] \, r[s,a], \end{aligned}$$

with *r*[*s*, *a*] as defined in (6). Note that agents in state ${\texttt{T}}$ choose action *a* with probability specified by $\pi [a \mid {\texttt{S}}]$ which coincides with $\pi [a \mid {\texttt{A}}]$ and $\pi [a \mid {\texttt{U}}]$ as assumed earlier. Similarly, for states $s, s^+ \in {\mathcal {S}}'$, the effective state transition matrix is defined as

7 $$\begin{aligned} \widehat{P}[s^+ \mid s](\pi ,\widehat{d}) = \sum _{a \in {\mathcal {A}}} \pi [a \mid s] \, \widehat{{\mathbb {P}}}[s^+ \mid s,a](\pi ,\widehat{d}). \end{aligned}$$

Assuming that the social state would not change, the expected discounted infinite horizon reward of an agent in state *s* with discount factor $\alpha \in [0,1)$ following the homogeneous policy $\pi$ is recursively defined as

$$\begin{aligned} V[s](\pi ,\widehat{d}) = R[s](\pi ) + \alpha \sum _{s^+ \in {\mathcal {S}}'} \widehat{P}[s^+ \mid s](\pi ,\widehat{d}) \, V[s^+](\pi ,\widehat{d}), \end{aligned}$$

or, equivalently in vector form,

8 $$\begin{aligned} V(\pi ,\widehat{d}) = (I - \alpha \, \widehat{P}(\pi ,\widehat{d}))^{-1} \, R(\pi ), \end{aligned}$$

which is the well-known Bellman equation. Note that for $\alpha \in [0,1)$, $I - \alpha \, \widehat{P}(\pi ,\widehat{d})$ is guaranteed to be invertible and accordingly, $V(\pi ,\widehat{d})$ is continuous in the social state $(\pi ,\widehat{d})$.

While an agent can compute the expected discounted reward $V(\pi ,\widehat{d})$ at a given social state $(\pi ,\widehat{d})$, the policy $\pi$ may not be optimal for the agent. We define the single-stage deviation value [20, Section 2.7] for an agent in state *s* choosing an action *a* for the present time step and subsequently following the homogeneous policy $\pi$ as

9 $$\begin{aligned} Q[s,a](\pi ,\widehat{d}):= r[s,a] + \alpha \sum _{s^+ \in {\mathcal {S}}'} \widehat{{\mathbb {P}}}[s^+ \mid s,a](\pi ,\widehat{d}) \, V[s^+](\pi ,\widehat{d}), \end{aligned}$$

i.e., the agent is aware of the immediate reward and the effect of its action on their future state; however, it assesses the future reward based on a stationarity assumption on $(\pi ,\widehat{d})$. In other words, the agent chooses its action to maximize a single-stage deviation from the homogeneous policy $\pi$, and assumes that its own actions are not going to affect the social state significantly. This assumption is fairly standard in the context of population games.

## Equilibrium Analysis

Having introduced the above model of state transitions and agent behavior, we here define and characterize the notion of stationary Nash equilibrium. Throughout this section, we assume that agents are aware of the true state distribution, i.e., $\widehat{d} = d$. We start by introducing the notion of best response map based on the single-stage deviation reward defined in (9).

The best response map at the social state $(\pi ,d)$ is the set valued correspondence $B: \Pi \times {\mathcal {D}}\rightrightarrows \Pi \subseteq \Delta ({\mathcal {A}})^{|{\mathcal {S}}|}$ given by

10 $$\begin{aligned} B(\pi ,d):= &   \Big \{\{\sigma _s \in \Delta ({\mathcal {A}})\}_{s \in {\mathcal {S}}} \mid \sum _{a \in {\mathcal {A}}} \left( \sigma _s[a] - \sigma '_s[a] \right) \, Q[s,a](\pi ,d) \ge 0, \nonumber \\  &   \quad \forall \sigma '_{s} \in \Delta ({\mathcal {A}}), s \in \{ {\texttt{S}}, {\texttt{I}}, {\texttt{R}}\}, \sigma _{\texttt{S}}= \sigma _{\texttt{A}}= \sigma _{\texttt{U}}. \Big \}. \end{aligned}$$

We have denoted the *Q* function for agents in superstate ${\texttt{T}}$ by $Q[{\texttt{S}},\cdot ]$ in the above equation with a slight abuse in notation in order to avoid introducing additional variables. Thus, the best response map is a correspondence from the space of social states to the set of policies for each epidemic state satisfying two properties:

- the policy for agents in states ${\texttt{S}}, {\texttt{A}}$ and ${\texttt{U}}$ coincide, and
- $B(\pi ,d)$ contains all randomized (mixed) strategies $\sigma$ over the actions that maximize expected single-stage deviation reward *Q* at the current state $s \in \{ {\texttt{S}}, {\texttt{I}}, {\texttt{R}}\}$ assuming that all other agents follow the homogeneous policy $\pi$ and their states are distributed as per *d*.

Since the set of actions is finite, there always exists a randomized strategy that maximizes the reward *Q* at every state, and hence the correspondence *B* is guaranteed to be non-empty. We now formally define the notion of a stationary Nash equilibrium to be a social state $(\pi ^*, d^*)$ such that the state distribution $d^*$ is stationary under the current state and policy $\pi ^*$, and $\pi ^*$ is a best response to the social state $(\pi ^*, d^*)$.

### Definition 1

(Stationary Nash Equilibrium) A stationary Nash equilibrium is a social state $(\pi ^*, d^*) \in \Pi \times {\mathcal {D}}$ which satisfies

SE.1 $$\begin{aligned} \pi ^*&\in B(\pi ^*, d^*), \end{aligned}$$

SE.2 $$\begin{aligned} d^*&= P(\pi ^*, d^*)^\top d^*, \end{aligned}$$

where $B(\pi ^*, d^*)$ is the best response map defined in (10) and $P(\pi ^*, d^*)$ is the stochastic matrix as defined in (1).

Thus, at the equilibrium, the stochastic matrix $P(\pi ^*, d^*)$ (1) is time-homogeneous, and the agents behave optimally in the Markov decision process defined by this stationary matrix [20]. Since the game considered here has a finite number of states and actions, and the state transition kernel and reward functions are continuous in the social state, it follows from [19] that a stationary Nash equilibrium exists in our setting.

### Theorem 1

(Theorem 1 in [19]) A stationary Nash equilibrium $(\pi ^*, d^*)$ for the proposed dynamic population game is guaranteed to exist.

Furthermore, it is easy to see that any stationary Nash equilibrium is infection free since both asymptomatic and symptomatic compartments are transient states for the transition probability matrix $P(\pi , d)$ irrespective of the social state.

## Social State Dynamics

Since the stationary Nash equilibrium is disease-free for this class of epidemics, it is critical to investigate the *transient* evolution of the state trajectory as well as the policy leading to the equilibrium.

The state update is governed by the time-varying transition probability matrix $P(\pi ,d)$. For the policy update, we get inspiration from the *evolutionary dynamic* models in classical population games [46], and consider the *perturbed best response dynamics*. In particular, we assume that the agents are not perfectly rational, with the bounded rationality factor $\lambda \in [0,\infty )$. When they are making a decision on which action to play, they follow the *logit choice* function [46, Sect. 6.2], given by

$$\begin{aligned} {\tilde{\pi }}[a \mid s](\pi ,d) = \frac{\exp {(\lambda \, Q[s,a](\pi ,d))}}{\sum _{a'} \exp {(\lambda \, Q[s,a'](\pi ,d))}}. \end{aligned}$$

It is evident that $\tilde{\pi }[\cdot \mid s](\pi ,d)$ is a probability distribution over the actions. For $\lambda = 0$, it results in a uniform distribution over all the actions, i.e., agents are not strategic and pick all available actions with equal probability. At the limit $\lambda \rightarrow \infty$, we recover the perfect best response. At finite values of $\lambda$, $\tilde{\pi }$ assigns higher probabilities to actions with higher payoffs.^1^

The combined policy-state distribution dynamics in continuous-time can be stated as

11 $$\begin{aligned} \dot{\pi }[\cdot | s]&= \eta _\pi \, \left( {\tilde{\pi }}[\cdot \mid s](\pi ,d) - \pi [\cdot \mid s](\pi ,d)\right) , \end{aligned}$$

12 $$\begin{aligned} \dot{d}&= P(\pi ,d)^\top d - d, \end{aligned}$$

where the parameter $\eta _\pi > 0$ controls the rate of policy changes with respect to the rate of social interactions. For $\eta _\pi < 1$, agents have *inertia* in their decision-making, i.e., policy changes occur at a slower time scale than interactions, and for $\eta _\pi = 1$, agents update their policy at the same time scale as state transition. When $\eta _\pi > 1$, policy update is faster than state distribution update.

In our numerical investigations in the following section, we consider discrete-time update equations given below

13 $$\begin{aligned} \pi _{k+1}[\cdot \mid s]&= \eta _\pi \eta _d \, {\tilde{\pi }}[\cdot \mid s](\pi _k,d_k) + (1 - \eta _\pi \eta _d) \, \pi _k[\cdot \mid s], \end{aligned}$$

14 $$\begin{aligned} d_{k+1}&= \eta _d P(\pi _k,d_k)^\top d_k + (1-\eta _d) d_k, \end{aligned}$$

where $\eta _d$ is the discretization parameter in the state distribution update equation. Note that this update model leads to a perturbed version of the Nash equilibrium policy $\pi$ at the rest points that captures bounded rationality in human behavior, rather than the exact policy [46].

## Numerical Results

We now illustrate the effectiveness of the proposed framework via numerical results. We present a select number of case studies to illustrate

- the effect of vaccination cost and availability limit on the evolution of infection state distribution and policy evolution of the agents,
- the impact of learning rate (timescale separation between state and policy update equations) on policy and state evolution, and
- the effectiveness of interventions (such as lockdown measures) on peak infection, aggregate activation, testing and vaccination under game-theoretic strategies by forward-looking agents.

Unless specified otherwise, we consider an infectious epidemic characterized by $\beta _{\texttt{A}}= 0.3$, $\beta _{\texttt{I}}= 0.2$, $\delta _{\texttt{A}}^{\texttt{I}}= 0.05$, $\delta _{\texttt{A}}^{\texttt{U}}= 0.05$, and $\delta _{\texttt{I}}^{\texttt{R}}=0.2$. We assume $\beta _{\texttt{A}}> \beta _{\texttt{I}}$ as it was observed during pandemics such as COVID-19 that individuals were most infectious just before the onset of symptoms [11, 50]. Furthermore, even if symptomatic individuals are more infectious and remain infected for a longer duration, yet they may cause less new infection due to being aware of their infection status and adopting suitable measures such as limiting their interactions. Consequently, we assume $\delta _{\texttt{A}}^{\texttt{U}}< \delta _{\texttt{I}}^{\texttt{R}}$.^2^ Each discrete time-step is assumed to represent one day.

We assume that agents can activate up to degree 5, i.e., ${a_{\text {max}}}=5$, and the activation reward is linear in the activation degree, with a unit reward for maximum activation $o[{a_{\text {max}}}] = 1$. The illness is quite severe, with a discomfort cost ${c_{\text {dis}}}= 7$. We assume that testing kits are not very expensive with ${c_{\text {test}}}= 0.1$. The parameter $\epsilon$ is set to be $10^{-3}$.

The agents are highly rational ($\lambda =20$) and forward-looking with discount factor $\alpha = 0.99$. The state distribution is updated with $\eta _d = 0.25$. For policy update, we have set $\eta _{\pi } = 0.5$, i.e., state distribution is updated at twice the rate of policy update. The initial state distribution is chosen such that $2\%$ of the population is asymptomatic (${\texttt{A}}$), $1\%$ is infected (${\texttt{I}}$) and the remaining are susceptible. Under the initial policy, infected agents do not activate while agents in other epidemic states choose to vaccinate with probability 0.01, undergo testing with probability 0.01 and choose an activation degree uniformly at random with the remaining probability.

We further consider that authorities can enforce lockdown regulations through the parameter ${a_{\text {lock}}}$, which represents the *maximum allowed activation degree*. Lockdown is implemented by setting $c[s,a] = 0$ if $a \le {a_{\text {lock}}}$, and $c[s,a] = 4 o[a]$ otherwise. Unless stated otherwise, we assume $c[{\texttt{R}},a] = 0$ for all *a*, i.e., we do not impose any restrictions on the activity of agents who are aware of their recovery. We set the activation cost for symptomatic agents in such a way that it is preferable for symptomatic agents to isolate themselves, i.e., $c[{\texttt{I}},0] < c[{\texttt{I}},a]$ for any activation degree $a > 0$. Thus, infections caused by asymptomatic agents is the primary factor behind the epidemic.

We emphasize that the above parameter values are chosen to illustrate a wide range of possible behavior that may emerge due to game-theoretic decision-making against infectious diseases in general (not necessarily limited to COVID-19). The exact numerical values may be changed to examine other possible scenarios or disease characteristics of interest (such as settings where symptomatic individuals are more infectious with slow recovery).

### Effect of Vaccination Cost and Availability

**Fig. 2:**
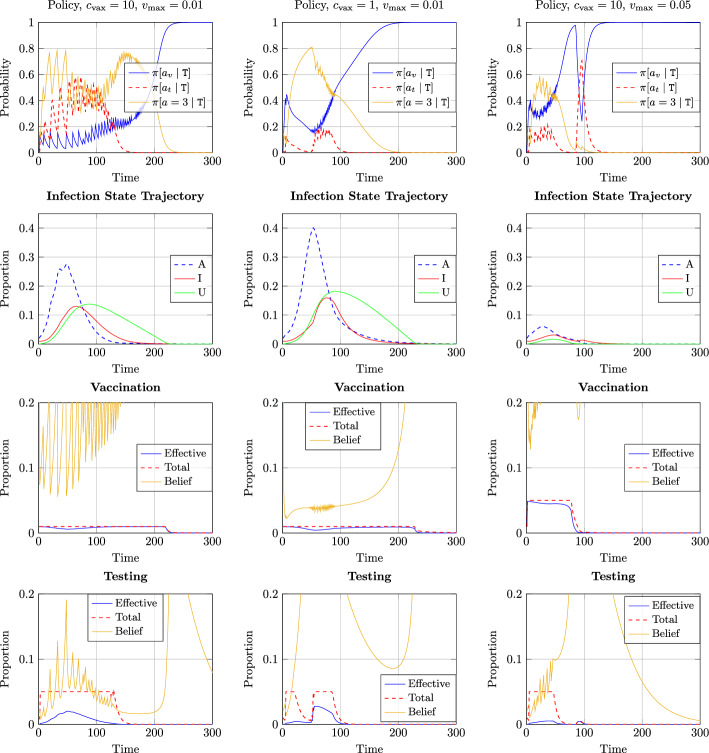
Evolution of vaccination, testing and activation policy for agents in state $\texttt{T}$ (top row), proportion of agents in states ${\texttt{A}}, {\texttt{I}}, {\texttt{U}}$ (second row), and quantities related to vaccination and testing (last two rows) with time for different choice of vaccination cost and availability limits (shown at the top of each column)

We first illustrate the effect of vaccination cost and availability in Fig. 2. We have assumed ${c_{\text {test}}}= 0.1, t_{\max } = 0.05$ for all figures. This choice of $t_{\max }$ implies that at most one twentieth of the entire population can undergo testing on a given day. For example, for several countries, the number of daily tests for COVID-19 per 1000 population hovered between 5 to 15 during year 2021 [12], and this rate is consistent with our choice of $t_{\max }$. The plots in the left panel correspond to the case where ${c_{\text {vax}}}= 10$ and $v_{\max } = 0.01$. The chosen value of $v_{\max }$ is consistent with the data on number of daily COVID-19 vaccine dose administration which hovered around $0.7-1\%$ of the population for many countries during 2021 [8, 13].

The plots in the middle panel correspond to the case where ${c_{\text {vax}}}= 1$ and $v_{\max } = 0.01$, i.e., vaccines are available at a cheaper cost. For the plots in the right panel, we have considered ${c_{\text {vax}}}= 10$ and $v_{\max } = 0.05$, i.e., more vaccines are available to be administered at each time step.

On the top row, we plot the evolution of the policy of agents in state ${\texttt{T}}$ over time for all three choices of vaccination cost and availability limit; specifically, the probability with which such agents choose to activate with degree ${a_{\text {lock}}}= 3$, choose to vaccinate and undergo testing. Since activation beyond ${a_{\text {lock}}}$ is severely penalized and activation with degree strictly smaller than ${a_{\text {lock}}}$ yields potentially smaller reward, those actions are chosen with negligible probabilities and hence not shown in the plots. The resulting infection state trajectories, i.e., proportion of agents in states ${\texttt{A}}, {\texttt{I}}$ and ${\texttt{U}}$ are shown in the second row of Fig. 2.

Note from the plots in the left panel that when vaccination cost is sufficiently high, agents in state ${\texttt{T}}$ choose vaccination with a smaller probability at the onset of the pandemic, and choose to activate or undergo testing otherwise. Such behavior is due to the fact that choosing to vaccinate is no guarantee to successfully obtain a vaccine due to limited availability. In fact, most of the population is susceptible at the onset of the pandemic, and a higher probability to vaccinate will lead to a greater proportion of agents opting for vaccination compared to vaccine availability, leading to a smaller probability of becoming successfully immune. As the infected proportion eventually reduces, agents in state ${\texttt{T}}$ begin to opt for vaccination with a greater probability. This is because as the proportion of agents in state ${\texttt{T}}$ is getting smaller, it is more likely that agents opting for vaccination will be successful in obtaining it (after which they move to state ${\texttt{R}}$ and obtain larger rewards by activating with ${a_{\text {max}}}$ as opposed to activating with ${a_{\text {lock}}}$ in state ${\texttt{T}}$). As a result, the proportion of agents in state ${\texttt{U}}$ gradually reduces as these agents move to state ${\texttt{R}}$ as observed in the state trajectory plots in the second row.

The plots on the first two rows of the left panel further show an oscillatory behavior of agents choosing to undergo activation or testing: a higher probability of activation leads to an increase in asymptomatic proportion which incentivizes agents to undergoing testing. On the other hand, a greater testing probability leads to more agents moving to symptomatic compartment from where they cause fewer infections ($\beta _{\texttt{I}}< \beta _{\texttt{A}}$) and also recover faster ($\delta _{\texttt{A}}^{\texttt{U}}< \delta _{\texttt{I}}^{\texttt{R}}$), leading to reduction in the infected proportions.

The results in the middle panel of the top row show that agents choose to vaccinate at a greater probability when vaccination cost decreases (${c_{\text {vax}}}= 1$). However, perhaps counter-intuitively, it leads to a significantly larger peak in the asymptomatic proportion (see plots in the left and middle panels of second row). This is due to the fact that vaccine availability has remained unchanged, and more agents opting to vaccinate does not result in any change in effective vaccinations. Rather, fewer agents opt for testing leading to ineffective isolation of asymptomatic, yet infectious, agents and a significantly larger peak in asymptomatic infection.

In order to obtain further insights on the impact of limited vaccine availability, we compare the following three quantities at each time step in the plots in the third row of Fig. 2:

- **total** mass of successful vaccination: $\min (v(\pi ,d),v_{\max })$ where $v(\pi ,d) = \sum _{s\in {\mathcal {S}}} d[s]\pi [a_v\mid s]$ is the total mass of agents who opt for vaccination at social state as defined earlier,
- **effective** vaccination, i.e.,
  $$\begin{aligned} \frac{\min (v(\pi ,d),v_{\max })}{v(\pi ,d)} (d[{\texttt{S}}] \pi [a_v |{\texttt{S}}] + d[{\texttt{U}}] \pi [a_v |{\texttt{U}}]), \end{aligned}$$
  which captures the proportion of agents that successfully transit to state ${\texttt{R}}$ due to vaccination, and
- the **belief** of an agent in state ${\texttt{T}}$ regarding the probability with which it will be successful in obtaining the vaccine and becoming immune if it chooses to vaccinate, i.e., the quantity
  $$\begin{aligned} \frac{\max (\widehat{d}[{\texttt{S}}],\epsilon ) + \widehat{d}[{\texttt{U}}]}{\max (\widehat{d}[{\texttt{S}}], \epsilon ) + \widehat{d}[{\texttt{A}}] + \widehat{d}[{\texttt{U}}]} \frac{\min (v(\pi ,\widehat{d}),v_{\max })}{v(\pi ,\widehat{d}) }. \end{aligned}$$

These plots corroborate the above discussion. Effective and total vaccination coincide most of the time except when asymptomatic proportion is significant compared to the total mass of susceptible and unknowingly recovered agents. The belief of an agent in state ${\texttt{T}}$ regarding the probability of successful vaccination increases with decline in the asymptomatic proportion.

The plots in the fourth row of Fig. 2 show the analogous of the above three quantities for testing. At the onset of the pandemic, as the asymptomatic proportion grows, agents in state ${\texttt{T}}$ choose to undergo testing and available testing kits are mostly exhausted. Nevertheless, since testing does not change the states of susceptible or unknowingly recovered agents, and the proportion of asymptomatic agents is much smaller compared to susceptible or unknowingly recovered agents, only a small fraction of tests are effective (i.e., lead to successful state transition from ${\texttt{A}}$ to ${\texttt{I}}$). The plots also show that testing is most effective when asymptomatic proportion is larger. Although testing results in asymptomatic agents incurring cost ${c_{\text {dis}}}$ in state ${\texttt{I}}$, such agents eventually recover, activate with degree ${a_{\text {max}}}$ and derive a larger reward compared to remaining in state ${\texttt{T}}$ and activating with degree ${a_{\text {lock}}}$ instead. Thus, testing remains an attractive option, particularly when vaccination cost is large and vaccine supply is limited. When vaccination cost reduces (plots in the middle panel) or vaccine availability is larger (plots in the right panel), testing becomes a less attractive option. Similarly, when the asymptomatic proportion decreases, testing is no longer an optimal action and agents choose to vaccinate with a larger probability.

### Effect of Learning Rate

**Fig. 3:**
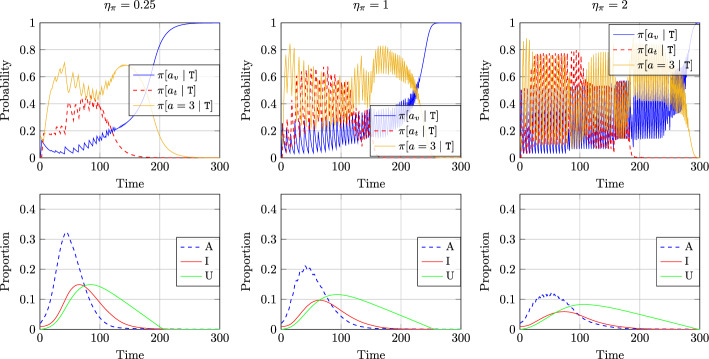
Impact of evolutionary learning rate $\eta _{\pi }$ on policy and state evolution of agents in state ${\texttt{T}}$ when state distribution is updated at rate $\eta _d = 0.25$

Figure 3 shows the impact of evolutionary learning rate $\eta _{\pi }$ on policy and state evolution of agents. We have kept the state distribution update rate parameter $\eta _d = 0.25$ and varied the parameter $\eta _{\pi }$. Recall that when $\eta _{\pi } = 1$, both state and policy update takes place at the same rate $\eta _d$ while for $\eta _{\pi } > 1$ (respectively, $\eta _{\pi } < 1$), policy update is faster (respectively, slower) compared to state distribution update. The plots show that when $\eta _{\pi } = 0.25$, the policy update takes place at a slower speed which leads to a smooth variation of the probabilities with which agents choose a certain action. As $\eta _{\pi }$ increases, the policy changes faster leading to more oscillations in these probabilities at the onset of the epidemic. The oscillations are specifically more pronounced when $\eta _{\pi } \ge 1$. As the epidemic dies out, the state distribution is nearly stationary and then the policy evolution becomes smooth, eventually converging to the stationary values. Note that the corresponding plots for $\eta _{\pi } = 0.5$ were shown in the left panel of Fig. 2 discussed earlier.

The plots in the second row of Fig. 3 show the corresponding evolution of proportion of individuals in asymptomatic, symptomatic and unknowingly recovered states. The results show that despite increased oscillations in the policy space, a larger $\eta _{\pi }$ results in a smaller peak infection, i.e., when agents frequently revise their policies, it leads to better outcome toward reducing peak infection level of the epidemic. The time at which peak asymptomatic infection is observed does not vary significantly with the learning rate.

The oscillations on the top row are primarily due to the coupled nature of evolutionary learning and epidemic dynamics. Similar oscillations were reported in recent works such as [33, 47] with myopic agents and more broadly in the context of feedback interconnection of optimization algorithms and dynamical systems [24, 25]. The relative rate of learning dynamics and plant (in this case, the epidemic model) dynamics plays a major role in the transient as well as asymptotic behavior of such coupled dynamics, which we plan to explore further in future work.

### Insights into Effectiveness of Lockdown Measures

**Fig. 4:**
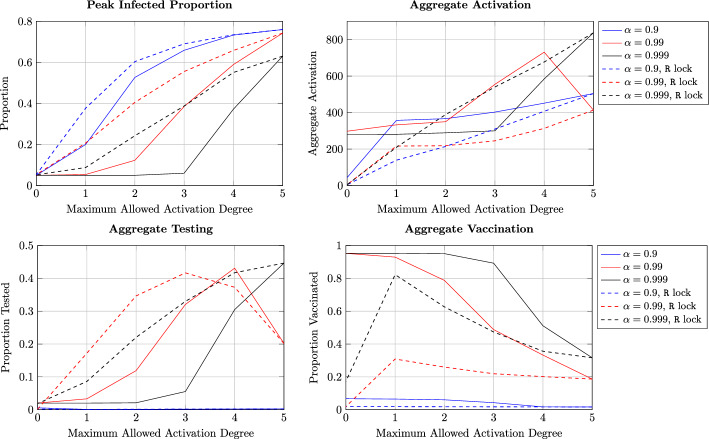
Aggregate outcome of game-theoretic decisions made by myopic and far-sighted agents under different types of social restrictions imposed by authorities

We now investigate the aggregate outcome of game-theoretic decisions made by myopic and far-sighted agents under different types of social restrictions imposed by authorities. In particular, we vary the parameter ${a_{\text {lock}}}$ from 1 to the maximum degree ${a_{\text {max}}}$ and plot the following four quantities under the dynamically evolving policy and population states in Fig. 4:

- the peak total infection (peak of sum of asymptomatic and infected proportions)
- aggregate activation (sum of aggregate activation by all agents in all states over the entire duration)
- aggregate testing (sum of effective mass of successful tests carried out over the entire duration), and
- aggregate vaccination (sum of effective mass of successful vaccination carried out over the entire duration).

The last three quantities are multiplied by the parameter $\eta _d$ to normalize them. We have used $v_{\max } = 0.01, t_{\max } = 0.05, {c_{\text {vax}}}= 10$ and ${c_{\text {test}}}= 0.1$ for the results in this subsection.

Recall that activation with degree below ${a_{\text {lock}}}$ does not incur any cost while activation with degree larger than ${a_{\text {lock}}}$ leads to severe penalty for this agent. Consequently, a smaller value of ${a_{\text {lock}}}$ signifies a more severe restriction on social interactions. As expected, the plots show that more stringent restrictions lead to smaller peak infection as well as activation.

We now compare the results for $\alpha = 0.9, 0.99$ and $\alpha =0.999$; the former sufficiently discounts future rewards while prioritizing stage rewards, while the agents are sufficiently far-sighted in case of $\alpha =0.999$. We also consider two further variations: when recovered agents are exempt from lockdown measures (shown in solid plots in Fig. 4) and when these agents remain subjected to lockdown measures despite being aware of their immunity status (shown in dashed plots).

The plots show that agents with $\alpha = 0.999$ sufficiently prioritize future rewards resulting in a significantly smaller peak infection and higher aggregate testing and vaccination. Nevertheless, aggregate activation by all agents does not exhibit significant difference for $\alpha = 0.9$ and $\alpha =0.999$. Thus, far-sighted agents are able to achieve a greater reduction in peak infection without sacrificing social interactions.

When activation restrictions continue to be imposed on recovered agents, we see a smaller degree of activation and vaccination by the agents (shown in dashed curves in the figure). In particular, since being aware of recovery does not lead to relaxation in social interactions, there are less incentives to vaccinate. However, sufficiently far-sighted agents choose to undergo testing with a slightly higher probability in this case. More interestingly, we observe that *peak infected proportion is slightly higher when recovered agents remain under restrictions compared to when these agents are exempt from restrictions* at all values of ${a_{\text {lock}}}$. This phenomenon is due to the fact that when recovered agents are exempt from social restrictions, they activate with degree ${a_{\text {max}}}$. Therefore, an activating susceptible agent is more likely to form connections with a recovered and immune agent compared to an asymptomatic or infected agent. When recovered agents are only allowed to activate with degree at most ${a_{\text {lock}}}$, there are two factors at play: (i) susceptible agents are less likely to vaccinate, and (ii) a susceptible agent is potentially more likely to get connected with an infectious agent leading to a higher probability of becoming infected.

## Discussions and Conclusion

We formulated a dynamic large population game from first principles to capture decision-making by individuals to protect themselves from an epidemic. In our formulation, agents choose whether to undergo testing, vaccination or to interact with other agents by examining the immediate reward as well as discounted future reward. Our work is one of the first to examine strategic considerations encountered by individuals as they choose (or avoid) to undergo testing using game theory.

We believe that our model has the minimal complexity required to capture human decision-making in presence of an epidemic. The presence of asymptomatic infectious agents necessitates adding additional compartments to the SIR model and adding testing as a possible action. Similarly, having vaccination as a possible action necessitates modeling agents to be forward-looking since the benefit of vaccination is only realized in the future. One common simplification in previous works has been to consider myopic agents but incorporate a heuristic term that depends on the risk of future infection in their myopic costs (e.g., [33, 47]). This term essentially substitutes the discounted future cost in our model (right term in (9)) and encompasses the future discount factor, the discomfort of illness, and the duration of illness all in one heuristic. While it could be possible to derive some analogous epidemiological insights with such a simplification, our model is more principled, interpretable, and allows for a finer grained analysis.^3^

In addition, the proposed framework naturally lends itself toward applying evolutionary learning strategies which enables us to investigate the joint evolution of infection states as well as agent decisions. We highlight the following key observations and policy implications obtained via our analysis and numerical results.

- Reducing the cost of vaccination without increasing its supply potentially leads to a higher level of peak infection because more agents opt for vaccination (as opposed to testing) while effective vaccination remains unchanged due to limited availability (Fig. 2).
- Continuation of restrictions on recovered (which includes vaccinated) agents potentially leads to reduced levels of vaccination and a greater peak infection, both for myopic as well as far-sighted agents (Fig. 4).
- When restrictions are withdrawn on recovered agents, both myopic and far-sighted agents exhibit similar level of total activity while peak infection is significantly smaller for far-sighted agents (Fig. 4).
- When policy update is faster than the rate of epidemic evolution, it leads to smaller peak infection, despite oscillatory behavior and frequent changes in the probabilities with which different actions are chosen (Fig. 3).

We conclude with a discussion on the following promising directions for future research.

- For several infectious diseases including COVID-19, recovered individuals are not permanently immune from future infection, rather they may get reinfected. However, including loss of immunity after recovery or vaccination in the epidemic model poses an important technical challenge. Specifically, a recovered or vaccinated individual does not know when it loses immunity, and thus, it does not know whether it is in state $\texttt{R}$ or state $\texttt{S}$. Thus, an individual is aware of its true state only when it is symptomatically infected. The super state $\texttt{T}$ would now include states $\texttt{S}$, $\texttt{A}$, $\texttt{U}$ and $\texttt{R}$ all of whom would follow the same policy. Such an assumption would be too simplistic to study game-theoretic decision-making. Another approach could be to allow an individual to keep track of its previous state and update its belief about loss of immunity as a function of how much time has passed since its last infection. However, this may render the decision problem non-Markovian. A more detailed modeling approach would be to consider partial observation in the dynamic population game framework. However, this is a theoretically challenging problem in itself. Thus, extending this work to include the possibility of reinfection and loss of immunity remains a challenging problem for future research.^4^
- While we have examined a specific class of evolutionary learning models in this work, other families of learning dynamics, such as the replicator dynamics, could also be examined.
- In this work, we assumed that parameters of the epidemic dynamics are known to the agents. However, this may not be true at the onset of an epidemic. While there have been much interest in multi-agent reinforcement learning in recent years, such strategies have not yet been explored in the context of dynamic population games in general and for epidemics in particular. Similarly, exploring evolutionary learning strategies for partially observed mean-field (epidemic) games remains a promising direction for future research.
- As observed during COVID-19, in several countries, intervention by authorities often failed to control the growth of the epidemic or caused severe economic damage as the response of the population was not accounted for. Indeed, we have highlighted several instances where counterintuitive outcomes may arise due to centralized interventions that are not so well thought out. Designing suitable intervention strategies that includes the response of the population requires extending the present framework to a Stackelberg (leader-follower) game formulation and analyzing learning strategies therein.

We hope this work stimulates further research along the above lines.

## Data Availability

All numerical results in the paper are obtained via simulations using parameters mentioned in the paper. No part of the paper uses any external dataset.

## Footnotes

1 In normal form games, an analogous model of human decision-making has been studied extensively in the past as it accounts for bounded rationality prevalent in human behavior by suitable choice of parameter $\lambda$. The corresponding notion of equilibrium is referred to as “Quantal Response Equilibrium” [22, 38].

2 From the perspective of epidemic state evolution, a symptomatic individual who is in strict isolation and is not able to cause new infection is analogous to a recovered agent.

3 For instance, it is challenging to include reversible actions such as activity level and one-shot actions such as vaccination simultaneously in a game involving myopic agents. Indeed, none of the prior works have analyzed strategic choice among vaccination, testing and activity levels simultaneously in the past. Nevertheless, individuals had to evaluate the trade-off among such possible actions during COVID-19.

4 Nevertheless, if we account for re-infections, we expect that the steady-state predictions of our model are likely to change, but we do not expect the crucial early stage of the outbreak to change significantly. Hence, our model provides important insights during the initial outbreak even for diseases imparting temporary immunity.
